# Nanoparticle Self-Assembled Grain Like Curcumin Conjugated ZnO: Curcumin Conjugation Enhances Removal of Perylene, Fluoranthene, and Chrysene by ZnO

**DOI:** 10.1038/srep24565

**Published:** 2016-04-15

**Authors:** Rasha N. Moussawi, Digambara Patra

**Affiliations:** 1 Department of Chemistry, American University of Beirut, Beirut, Lebanon

## Abstract

Curcumin conjugated ZnO, referred as Zn(cur)O, nanostructures have been successfully synthesized, these sub-micro grain-like structures are actually self-assemblies of individual needle-shaped nanoparticles. The nanostructures as synthesized possess the wurtzite hexagonal crystal structure of ZnO and exhibit very good crystalline quality. FT-Raman and TGA analysis establish that Zn(cur)O is different from curcumin anchored ZnO (ZnO@cur), which is prepared by physically adsorbing curcumin on ZnO surfaces. Chemically Zn(cur)O is more stable than ZnO@cur. Diffuse reflectance spectroscopy indicates Zn(cur)O have more impurities compared to ZnO@cur. The solid-state photoluminescence of Zn(cur)O has been investigated, which demonstrates that increase of curcumin concentration in Zn(cur)O suppresses visible emission of ZnO prepared through the same method, this implies filling ZnO defects by curcumin. However, at excitation wavelength 425 nm the emission is dominated by fluorescence from curcumin. The study reveals that Zn(cur)O can remove to a far extent high concentrations of perylene, fluoranthene, and chrysene faster than ZnO. The removal depends on the extent of curcumin conjugation and is found to be faster for PAHs having smaller number of aromatic rings, particularly, it is exceptional for fluoranthene with 93% removal after 10 minutes in the present conditions. The high rate of removal is related to photo-degradation and a mechanism has been proposed.

ZnO occurs as the rare mineral zincite, usually appearing as a white powder, it’s poorly soluble in neutral water but soluble in acids and alkali. It crystallizes in three different structures^1^: wurtzite, zinc blende and rock salt. The wurtzite structure of ZnO is the thermodynamically stable phase; the two other structures of ZnO, the zinc blende and the rock salt are metastable and only occur under certain conditions. The zinc blende structure is obtained through epitaxial growth of ZnO on a suitable cubic substrate, while the rock salt (NaCl-type) structure is observed when subjected to high pressures (~9 GPa at 300 K). Nearly all photo-catalytic studies are focused on the wurtzite structure.

Perylene, fluoranthene, chrysene etc. are polycyclic aromatic hydrocarbons (PAHs) and at the top of the pollutants list of US EPA and European Union, due to their toxic, mutagenic, and carcinogenic potential^2^. These compounds are introduced to the environment through natural and anthropogenic processes^3^. Ascomycota fungi are proposed to be the major precursor carriers for perylene in sediment^4^. Anthropogenic sources of such compounds include oil spills from crude and refined petroleum, introduced to aquatic environments through accidental discharge from tanks and municipal and urban pipes^5^,^6^,^7^. These are also produced by incomplete combustion of fossil fuels^8^,^9^,^10^,^11^. Smoking, gas cooking, unvented kerosene heating, and heating appliances can be significant sources in indoor air^12^,^13^. PAHs contaminated in water can be processed efficiently by adsorption with activated carbon or other adsorbents, or by conventional chemical treatments^14^. However, these procedures have failed to achieve the purity required by law in certain cases and conditions. One of the major transformation processes resulting in the degradation of PAHs in water is photo-catalytic oxidation process. These photo-catalytic oxidation processes use semiconductor materials (ZnO and TiO_2_) for the removal of the residual concentrations of several PAHs from water below the standard levels. Advantages of the photo-catalytic process over other techniques include its mild operating conditions and its reliance on sunlight as the power source, thus significantly reducing the operating costs of electric power required.

Although many semiconductors such as TiO_2_, WO_3_, ZrO_2_, SnO_2_, Fe_2_O_3_, CdS, ZnS, WS_2_, MoS_2_ have been tested for the photo-catalytic degradation of various environmental contaminants^1^, an advantage of ZnO is its low cost and that it absorbs over a larger portion of the solar spectrum^15^. For this reason, ZnO is considered the most suitable catalyst for photo-catalytic removal in the presence of sunlight. Since the minimum energy required for excitation of an electron for ZnO is 3.2 eV (which corresponds to a UV wavelength of ~387.5 nm) and solar light contains less than 4% of UV light, the application of ZnO is limited. To expand the use of ZnO photo-response to the visible region, surface modification and dye sensitization are effective methods. Kou *et al.*^16^ showed that GaN:ZnO has excellent properties for the photo-degradation of PAHs.

Curcumin accounts as the major component (approx. 77%) in the curcuminoid present in turmeric^17^. Curcumin is well known for its pharmaceutical applications and medicinal potential in therapy of many diseases^18^,^19^,^20^. Reports on curcumin as sensitizer are limited. Curcumin-derived dye can be used as a sensitizer in dye sensitized solar cells^21^ and it has very exciting fluorescence properties as a probe^22^,^23^,^24^ and sensing^25^,^26^ molecule. Curcumin forms metal complex with various metal ions^27^,^28^,^29^. Curcumin acts as a mild reducing agent to prepare Ag nanoparticles^30^ and Au nanorods^31^. It also makes a Zn-curcumin complex by reacting with zinc salt^32^,^33^. Since zinc can form complexes with curcumin through its β-diketo group and presence of curcumin on zinc oxide surfaces could increase adsorption of PAHs on ZnO surfaces (due to the hydrophobic nature of curcumin as well as through π-stacking between curcumin and PAHs), in this work we prepared curcumin conjugated zinc oxide (Zn(cur)O) where curcumin was incorporated with ZnO during the synthesis process through a wet chemistry method. The materials were characterized by spectroscopic and other techniques. The morphology of curcumin conjugated ZnO nanostructures was found to be sub-micro grain-like structures, which are due to self-assembly of needle-like nanoparticles. The nanostructures synthesized possessed the wurtzite hexagonal crystal structure of ZnO and showed very good crystalline quality. In line with exploring the environmental applicability of Zn(cur)O as a detoxifying material, the removal capacity of the compound was tested on perylene, fluoranthene, and chrysene. Removal rate by Zn(cur)O was compared with naked ZnO, where Zn(cur)O was found to be more efficient than bare ZnO. The high rate of removal was related to photo-degradation and a mechanism has been proposed.

## Materials and Methods

### Materials

Zinc nitrate hexahydrate (Zn(NO_3_)_2_·6H_2_O)(98% extra pure) was obtained from Acros Organics. Curcumin was received from Sigma. Potassium hydroxide was sourced from AnalaR. Acetone (HPLC grade), perylene, chrysene and fluoranthene were received from Sigma-Aldrich. All the solutions were prepared with deionized water unless otherwise mentioned.

### Synthesis

Zn(cur)O was prepared by taking 0.5 mg, 1.0 mg, or 1.5 mg of curcumin in 50 mL of doubly distilled water (close to neutral pH) at 80–90 ^o^C. When curcumin was solubilized completely, 50 mL of 0.1 M Zn(NO_3_)_2_ solution prepared in doubly distilled water was added to it. The yellowish solution was refluxed for 1 hour at 85-90 ^o^C. After that, the solution was cooled down and 5 mL of 0.2 M KOH was added slowly at 4 ^o^C. An orange yellow gel-like suspension was observed. The solution was centrifuged at 5000 rpm and the precipitate was washed with water till no more yellow color was observed in the supernatant. Acetone washes were necessary to remove any unreacted curcumin after which the final wash was with water. The precipitate Zn(cur)O was vacuum dried at room temperature. A control ZnO was synthesized by taking 50 mL of 0.1 M Zn(NO_3_)_2_ solution prepared in doubly distilled water and then adding 5 mL of 0.2 M KOH slowly in an ice bath at 4 ^o^C. The white suspension was centrifuged at 5000 rpm, washed with water and vacuum dried just like Zn(cur)O. Similarly, curcumin anchored ZnO (ZnO@cur) was prepared by mixing curcumin with the ZnO in solutions, where curcumin and ZnO are connected physically, i.e., via static interactions, van der Waals forces or Lewis acid–base interactions. In the case of ZnO@cur, washing with acetone could easily removed curcumin from the ZnO surfaces.

### Characterization

The apparent zeta potential was measured using a Malvern Zetasizer Nano ZS (M3-PALS) using the Non-Invasive Back Scatter technique. The instrument was equipped with a monochromatic red laser operating at 632.8 nm and the data were analysed with the Malvern Dispersion technology software. Z-average values for three measurements were recorded. The Thermogravimetric Analysis (TGA) and Differential Scanning Calorimetry (DSC) measurements were done using a Netzsch TGA 209 in the temperature range 0 to 800 ^o^C with an increment of 30 K/10 minutes in a N_2_ atmosphere. Scanning electron microscopy (SEM) analysis was carried out using Tescan, Vega 3 LMU with Oxford EDX detector (Inca XmaW20) SEM. The sample was deposited on a carbon film for SEM analysis. Transmission electron microscopy (TEM) measurement was carried out with a JEOL 2200FS double aberration corrected FEG microscope, operating at 200 kV. TEM samples were prepared by casting a drop of the nanoparticle suspension onto copper grids covered with holey carbon films. The X-Ray Diffraction (XRD) data were recorded using a Bruker d8 discover X-Ray diffractometer equipped with Cu-Kα radiation (λ = 1.5405 Å). The monochromator used was Johansson Type. The step size was 0.02 degree and the scan rate was 20 s per step.

### Spectroscopic analysis

The chemical structures of curcumin, Zn(cur)O and, ZnO were characterized by FT-Raman spectroscopy. The absorption spectra were recorded at room temperature using a JASCO V-570 UV-VIS-NIR Spectrophotometer. Similarly, UV-visible Diffuse Reflectance spectra were measured using the same spectrophotometer in the range 200 to 800 nm. The solid-state photoluminescence spectra were recorded at room temperature using Jobin-Yvon-Horiba Fluorolog III fluorometer and the FluorEssence program where the excitation and emission slits width were 5 nm. The source of excitation was a 100 W Xenon lamp, and the used detector was R-928 operating at a voltage of 950 V.

### Removal of PAHs

30 mL of 50 μM (12.6 mg/L) perylene was prepared from 1 mM stock solution (perylene in acetonitrile). 5 mg of Zn(cur)O was added into the pervious solution while stirring (167 mg/L). Simply letting air go through the erlenmeyer’s opening provided the O_2_ requirement. Immediately 5 mL of the solution was withdrawn after centrifuging or allowing the compound (Zn(cur)O) to settle down to make sure no Zn(cur)O was being withdrawn along. The separation was efficient as no absorption of curcumin was detected in the sample. The removal was studied by recording absorption spectra at room temperature. The absorbance of the sample withdrawn was monitored in different time interval (10–20 minutes interval) depending on the rate of removal. The same procedure was followed with fluoranthene (11.4 mg/L) and chrysene (11.1 mg/L). For all these experiment, day light conditions were maintained.

## Results and Discussion

### Morphological Characterization

**Figure 1:**
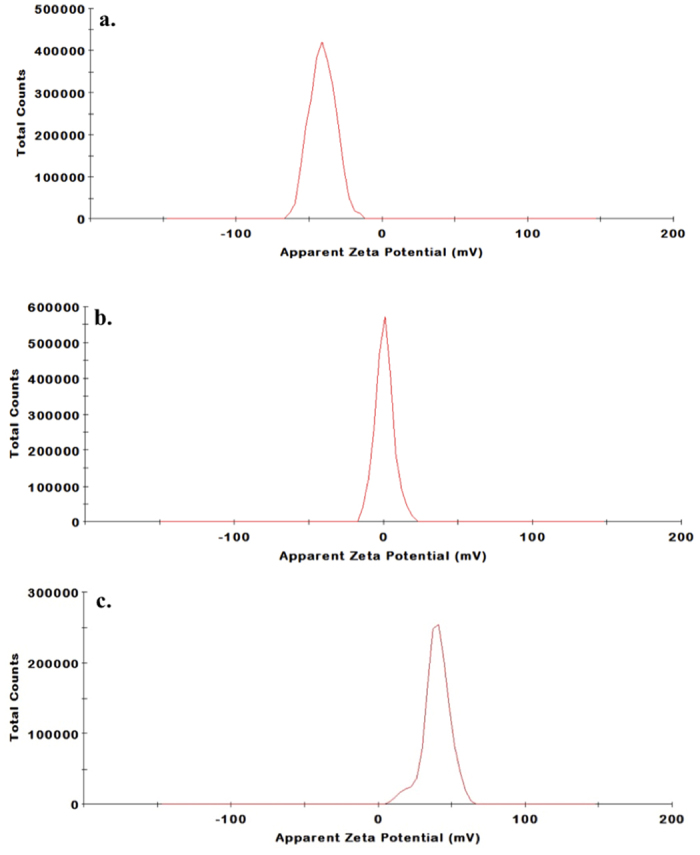
Apparent Zeta Potential distribution of (**a**) Curcumin, (**b**) ZnO and (**c**) Zn(cur)O.

**Figure 2:**
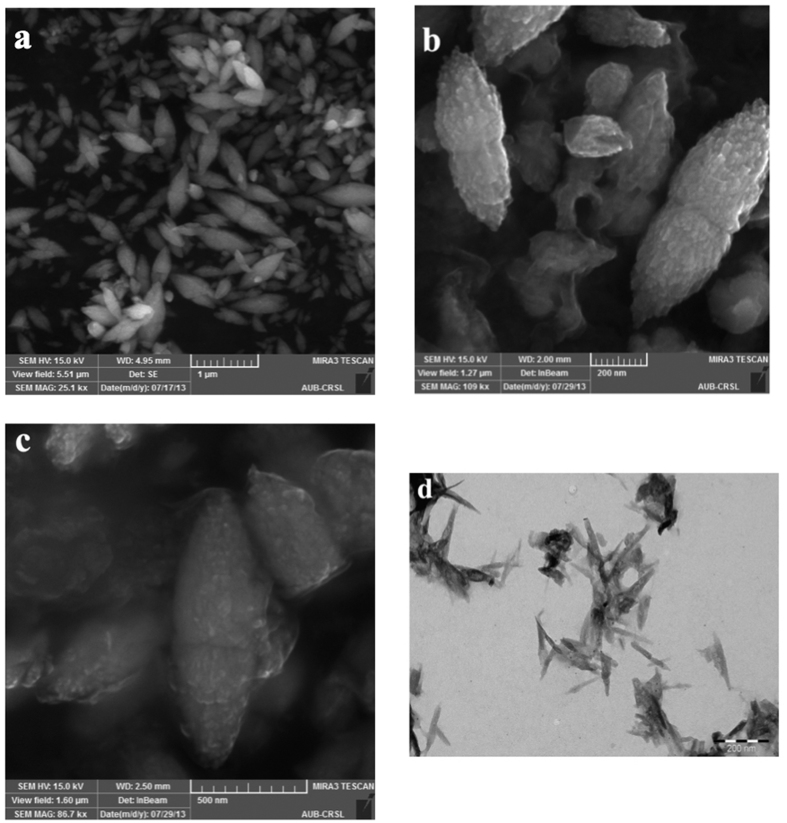
SEM images of (**a**) ZnO anchored with curcumin (ZnO@cur), (**b**) Zn(cur)O using 0.5 mg of curcumin and (**c**) Zn(cur)O using 1.5 mg of curcumin. (**d**) TEM image of Zn(cur)O after strong ultra-sonication.

The apparent zeta potential gives information related to surface charge. The measurements were done in doubly distilled water where the pH was 6.8–7. As depicted in Fig. 1 the zeta potential measurement of curcumin in water showed a value close to −40 mV whereas that of ZnO was found to be nearly 0 mV in water. This suggests ZnO is in neutral form in solution but the enolic form of curcumin dominates in solution (pK_a1_ of curcumin is close to 7.8). When curcumin was mixed with zinc nitrate, a zeta potential value slightly negative but close to zero was observed. This observation is not surprising as Zn^2+^ can form a complex with curcumin by neutralizing the negative surface charge of curcumin solution^33^. Now for Zn(cur)O, the zeta potential remarkably changed to +40 mV confirming that Zn(cur)O has a positive surface charge and is relatively stable reflecting the strong interaction between curcumin and ZnO. This is possible when Zn(II) coordination sphere is filled by intercalating two O, O chelating active ligands of curcumin. Morphologically, Zn(cur)O particles were found to have a grain-like structure. The particles size and shape are depicted in SEM images given in Fig. 2. Length of these particles was between 600 and 2000 nm and width was from 200–600 nm. However, smaller and broken particles were also observed. During preparation of Zn(cur)O, concentration of curcumin did not influence the morphology of the particles. Similarly, ZnO and anchoring of ZnO with curcumin (ZnO@cur) did not the change the appearance. Interestingly, similar structures of ZnO are reported in literature^34^. This suggests curcumin did not have any major influence in determining the morphology of the particles. To check whether these Zn(cur)O particles were assembled of nano-sized particles, the samples were subjected to strong ultra-sonication. Strong ultra-sonication destroyed these bundles to fragmented needle shaped nanoparticles as shown in Fig. 2d. These particles were found to be around 200 nm lengths and 10–20 nm in width. Similar observations were made for naked ZnO.

### XRD Measurements

**Figure 3:**
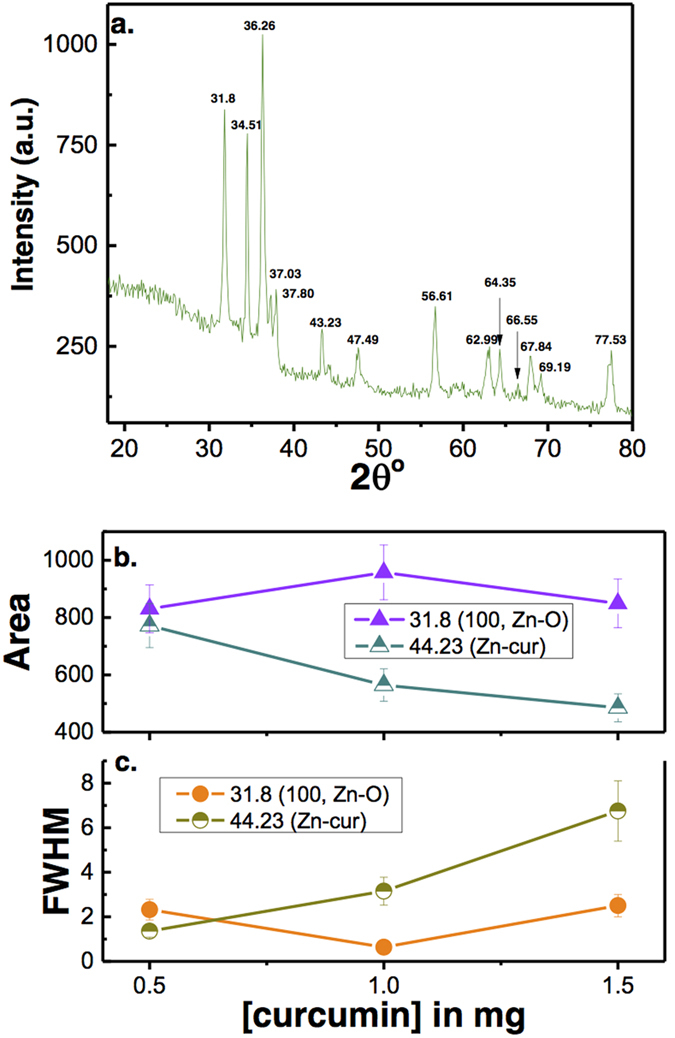
(**a**) X-ray diffraction patterns of Zn(cur)O nanoparticles as prepared using 1.5 mg of curcumin. (**b**) The change in X-ray peak area and FWHM at peak positions 31.8 (for Zn-O) and 44.23 (for Zn-cur) is plotted with curcumin concentration.

In the XRD pattern given in Fig. 3a, all the Zn-O hexagonal phase diffraction peak were found such as peaks at 31.80 (100), 34.51 (002), 36.26 (101), 47.49 (102), 56.61 (110), 62.99 (103), 66.55 (200), 67.84 (112), 69.19 (201) and 77.53 (202). These observed peaks are in good agreement with those for hexagonal ZnO with wurtzite structure as reported earlier^35^. However, the additional peaks were found at 37.03, 37.80, 43.23 and, 64.35, which are due to curcumin^36^. The Scherrer equation^37^ relates average crystalline size of the sample with the line broadening at full width at half maximum (FWHM). Thus, the influence of the curcumin concentration in diffractograms of X-ray diffraction of the samples can be understood in the variations of intensity and FWHM of the diffraction peaks of Zn(cur)O. These characteristics are useful to determine crystallinity degree and crystalline size of the sample. The peak at 31.8 was monitored to evaluate relative crystallite size and crystallinity with curcumin concentration used during synthesis of Zn(cur)O. It was found that as the curcumin concentration increased from 0.5–1 mg the crystalline size increased (a decrease in FWHM, see Fig. 3c) but further increase in curcumin concentration to 1.5 mg decreased the crystalline size. At the same time peak area initially decreased and then increased which is as expected opposite with variation in crystalline size (Fig. 3b). On the other hand the peak at 43.23 for Zn-curcumin was monitored, which decreased with curcumin concentration.

### FT-Raman Spectroscopic Analysis

**Figure 4:**
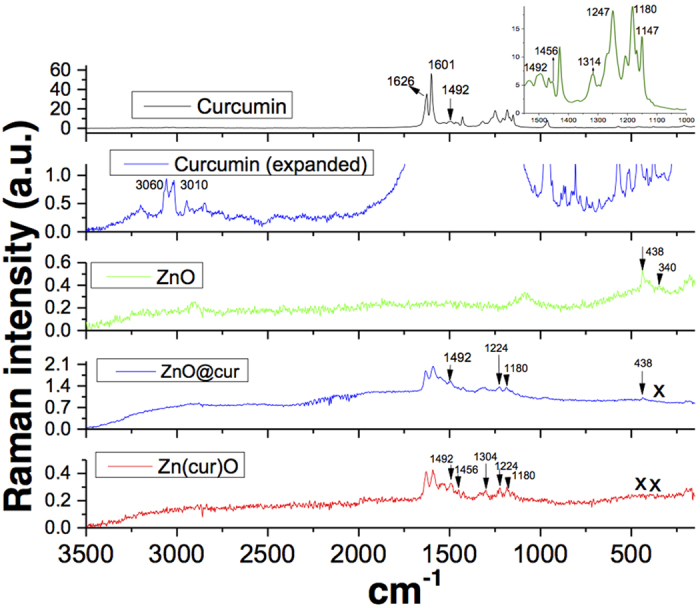
Raman spectra of as-synthesized Zn(cur)O, ZnO and ZnO@cur nanostructure materials at room temperature. The FT-IR spectrum of curcumin is shown for comparison.

As shown in Fig. 4, the observed Raman shift of curcumin at around 3060 and 3010 cm^−1^ are assigned to aromatic C-H_stretching_. The Raman shift at 1626 cm^−1^ is due to ν_C=C_ and ν_C=O_ of curcumin, which is same as reported to experimental value and close to computed values 1630 cm^−1^ and 1615 cm^−1^ respectively^38^. Absence of peaks in the region of 1650–1800 cm^−1^ further suggest the curcumin largely exists in enol form rather than keto form corroborating earlier findings^38^. The band at 1601 cm^−1^ and 1492 cm^−1^ (shown as arrow) is due to aromatic vibration ν_C = Cring_ of curcumin. All these bands were found in Zn(cur)O and ZnO@cur. However the δ_CH3_ band at 1456 cm^−1^ of curcumin shifted in ZnO@cur whereas it did not show any change in Zn(cur)O. The band at 1630 cm^−1^ is due to phenolic OH group which was also found in both Zn(cur)O and ZnO@cur. The band at 1314 cm^−1^ is due to δ_PhCCHOHenol_. The peak at 1304 cm^−1^ was missing in curcumin, which is for δ_COHenol_, but in the case of Zn(cur)O a Raman shift at around 1304 cm^−1^ was observed. The band at 1247 cm^−1^ due to δ_COHenol_ of curcumin also shifted to around 1224 cm^−1^ in Zn(cur)O and ZnO@cur indicating presence of CO type bond with zinc. The band at 1180 and 1147 cm^−1^ of curcumin due to δ_CH3_ and ν_O-CH3_ remained same in Zn(cur)O and ZnO@cur. For ZnO, the Raman-active phonons predicted by group theory are A_1_ + 2B_1_ + E_1_ + 2E_2_. The B_1_ (low) and B_1_ (high) modes are normally silent, A_1_, E_1_, and E_2_ are Raman-active and A_1_ and E_1_ are also infrared-active^39^. The E_2_ is a non-polar phonon mode with two frequencies of E_2_ (high) corresponding to oxygen atoms and E_2_ (low) corresponding to Zn. Both the A_1_ and E_1_ are polar phonon modes, thus they each experience frequencies for transverse-optical (TO) and longitudinal-optical (LO) phonons^39^. The dominant line at 438 cm^−1^ corresponds to the E_2_ (high) vibration mode, which is a characteristic band of wurtzite phase with orientation in the c-axis, is noticed in ZnO. The spectrum also shows the forbidden mode at 340 cm^−1^ (humps) frequency of second order described by E_2_(high) −E_1_ (low) phonons. The peaks at 580 cm^−1^ correspond to the A_1_ (LO) and E_1_ (LO) vibration modes, which indicate the crystal disorder if the peaks are shifted to a different frequency. The peak at 580 cm^−1^ is a combination of the two modes, thus very broad and enhanced by disorder^39^, though they remain at lower intensity due to more ordered wurtzite structures as seen in the peak at 438 cm^−1^. The E_1_ (LO) mode is theoretically not allowed according to the Raman rules, however it can be visible if the incident light beam direction is not well defined with the axis of the nanostructure (*c*-axis). The appearance of E_1_ (LO) also indicates oxygen deficiencies. The peaks at 380 and 410 cm^−1^ correspond to A_1_ (TO) and E_1_ (TO) respectively. These peaks are usually present due to the structural induced disorder in the ZnO substrate. However, ZnO@cur showed the peak at 438 cm^−1^ and did not show any other peaks. On the other hand none of the peaks including one at 438 cm^−1^ of ZnO were present in Zn(cur)O.

### Thermogravimetric Analysis

**Figure 5:**
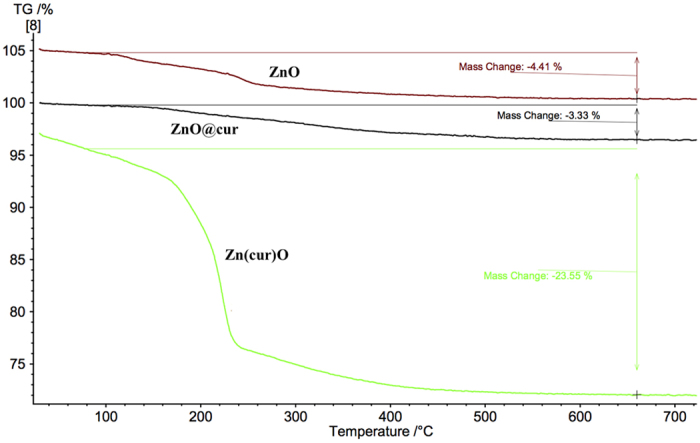
TGA of ZnO, ZnO@cur and Zn(cur)O.

**Figure 6:**
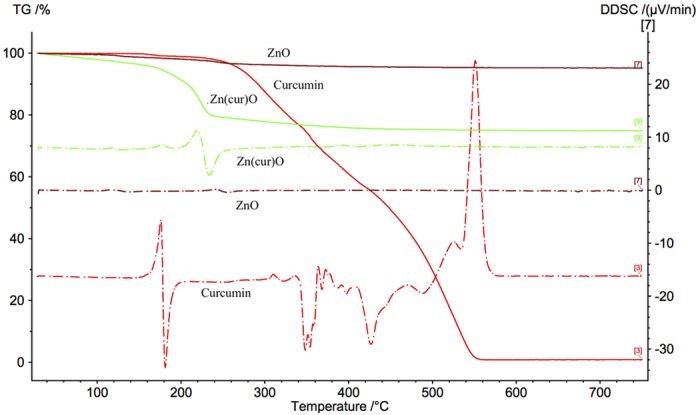
Derivative of DSC of curcumin, ZnO, ZnO@cur and Zn(cur)O compared with TGA of curcumin.

The TG–DSC curves of the precipitates Zn(cur)O, ZnO, ZnO@cur and raw curcumin are shown in Figs 5 and 6. The TG curve indicates that a remarkable mass loss (~23%) occurred during the thermal decomposition of Zn(cur)O and a range of 3–4% mass loss for ZnO and ZnO@cur. The precipitate Zn(cur)O began to decompose with temperature and when the temperature was above 180 °C it accelerated, and the decomposition was complete at ~240 °C. The weight-loss in raw curcumin occurs around 260 °C till around 550 °C, whereas the weight loss for ZnO occurred in the range 220**–**280 °C (Fig. 5). The derivative of differential scanning calorimetry (DSC) depicted in Fig. 6 showed a minimum at around 250 °C for ZnO. For raw curcumin the minima was found to be at around 180 °C and for Zn(cur)O at around 230 °C. The maxima at 160 °C for raw curcumin could be detected in Zn(cur)O in addition to maxima at around 200 °C due to the main weight loss of Zn(cur)O, but one of the major maximum at around 540 °C in raw curucmin was not found in Zn(cur)O. The small bump found at around 105 °C in Zn(cur)O is similar to one obtained in ZnO, this could be due to small amount water as impurities or measurement error. However, ZnO@cur showed TGA and DSC data similar to ZnO as revealed in Figs 5 and 6. Interestingly, weight loss in curcumin and ZnO@cur followed a gradual decrease despite weight loss was 100% for curcumin whereas in ZnO and Zn(cur)O the weight loss occurred more rapidly. This, along with the shift in peaks in thermogram of Zn(cur)O compared to ZnO clearly indicates that Zn(cur)O is different from ZnO anchored with curcumin (ZnO@cur) or ZnO.

### Optical Absorption Spectra

**Figure 7:**
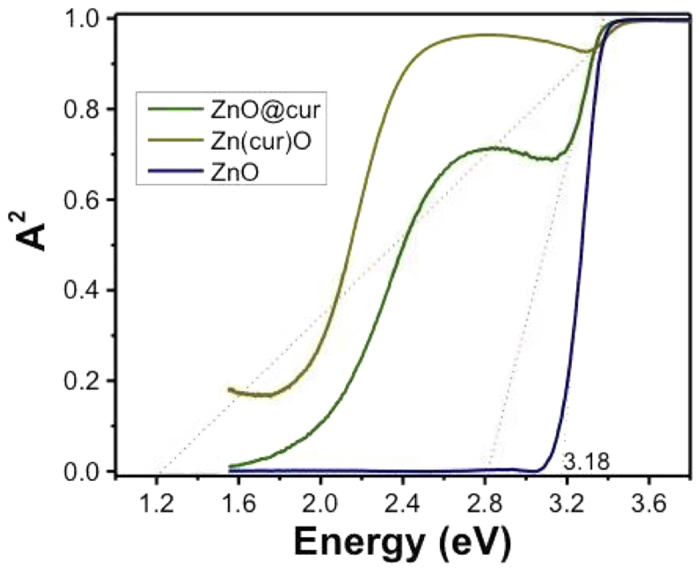
Plot of (A)^2^ versus the energy of absorbed light for the as-synthesized ZnO, Zn(cur)O and ZnO@cur nanostructures at room temperature. A^2^ is square of absorbance value.

Figure 7 shows a plot of A^2^ (square of absorbance value) versus the energy of absorbed light for ZnO, ZnO@cur and Zn(cur)O. From this plot the direct band gaps can be estimated to be 3.18 eV for ZnO as synthesized in our lab using the procedure explained in the materials and methods section, which is similar to the reported value for bulk ZnO (3.37 eV)^40^. Such broadening of band gap and similar value has been reported during doping of ZnO nanostructured materials by other metal^41^. The variation in band gaps is ascribed to the occurrence of shallow donor levels introduced by impurity atoms. Further, the direct band gap was estimated as 2.8 eV for ZnO@cur and 1.2 eV for Zn(cur)O indicating Zn(cur)O have more impurities atom (O**-**atom coming from curcumin) compared ZnO anchored with curcumin. It is worth noting that a band around 2.4–2.5 eV was observed in Zn(cur)O and ZnO@cur due to absorption of curcumin.

### Solid State Photoluminescence Study

**Figure 8:**
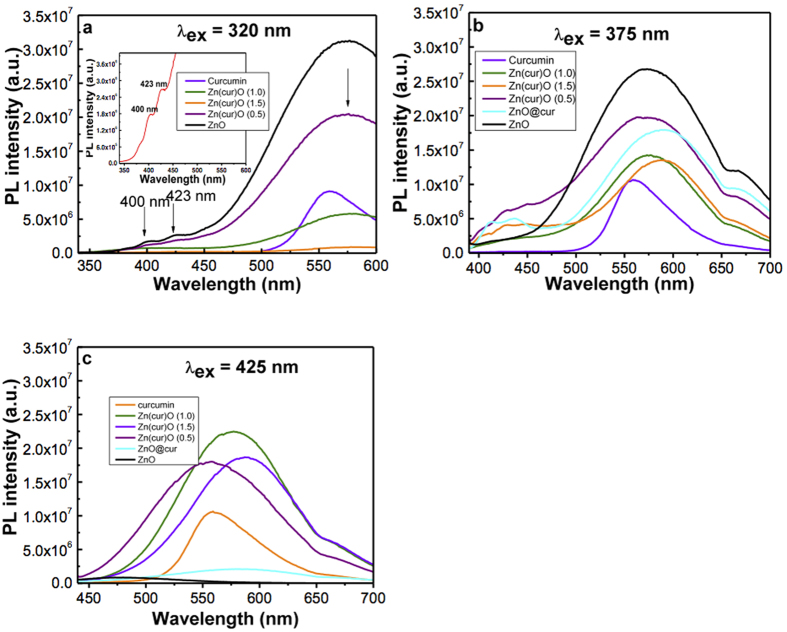
Solid state Photoluminescence spectra at room temperature of ZnO and Zn(cur)O at excitation wavelengths (**a**) 320 nm (**b**) 375 nm (**c**) 425 nm.

The solid state photoluminescence of powder Zn(cur)O was taken at different excitation wavelengths as given in Fig. 8. The solid state PL is different from PL in solution and this is expected as the solvent environment directly or indirectly influences PL spectra in solution due to defects, whereas self-quenching of materials may shift the position of the spectrum and/or quench PL intensity in the solid state. The visible PL at 575 nm for the excitation wavelength at 320 nm decreased in comparison to ZnO as the curcumin content was increased from 0.5 mg to 1.0 mg and 1.5 mg. The visible photoluminescence quenching in Zn(cur)O indicates that curcumin fills the defects of ZnO. The red shift of ZnO and Zn(cur)O visible PL compared to ethanolic solution (not shown) PL is expected because in the solid state the energy transfer or self-quenching is unavoidable. Two additional peaks at 400 nm and 423 nm were also found for ZnO, which decreased with the increase in curcumin content. For an excitation wavelength at 375 nm, it’s noticed that the 575 nm PL peak didn’t decrease substantially as before and the Zn(1.0cur)O and Zn(1.5cur)O peaks were almost similar in intensities, decreasing up to ~47% of the curcumin free ZnO intensity, except that Zn(1.5cur)O had a red shift by 10 nm, which is significant. At 425 nm excitation wavelength, a very weak peak at 475 nm was observed for ZnO. ZnO is expected not to have emission in this excitation wavelength region and curcumin fluorescence at this excitation wavelength is known^30^,^31^. Therefore, the emission at excitation wavelength 425 nm is dominated by curcumin emission. As for the Zn(cur)O, the PL varies due to contribution from curcumin, but the change in PL with curcumin content was found to be similar to what was observed at excitation 375 nm.

### Removal of Perylene, Chrysene and Fluoranthene

**Figure 9:**
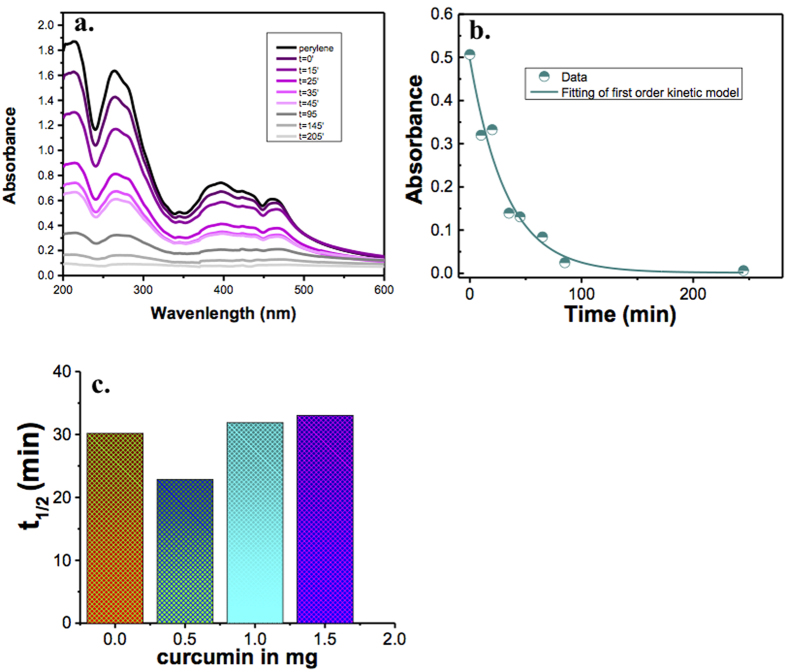
(**a**) UV-visible absorption spectra of perylene during degradation by Zn(cur)O; (**b**) Plot of change absorbance change with time, the fitting shown a first order rate equation: (**c**) Histogram showing half-life during perylene degradation by ZnO and Zn(cur)O for different curcumin concentration.

The removal capabilities of ZnO and Zn(cur)O were investigated using perylene. The removal of perylene (Fig. 9a) was monitored by UV-visible spectrophotometer for various time intervals after filtering out Zn(cur)O. As shown in Fig. 9a the absorbance of perylene continued to decrease with time indicating its removal. Note that the removal process is so fast and onsets as soon as Zn(cur)O is added to perylene as noted from the absorbance recorded just before any addition of Zn(cur)O and immediately after mixing and filtering out Zn(cur)O.

The change in absorbance with time during removal of perylene by Zn(cur)O is given in Fig. 9b. The data fitted well to a first order rate law as:


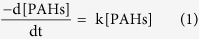


where *k* is the first order rate constant. The half-life of the reaction, which is independent of initial concentration, was used to compare activities of different Zn(cur)O and ZnO. The half-life was calculated as:


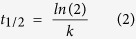


**Figure 10:**
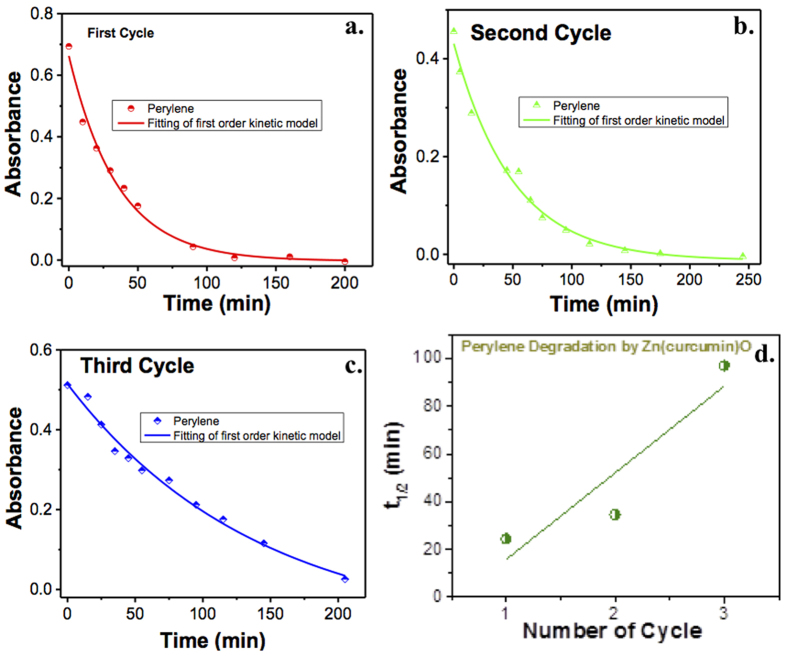
Change in absorbance of perylene during degradation by Zn(cur)O for (**a**) first cycle, (**b**) second cycle and (**c**) third cycle. (**d**) Plot of t_1/2_ versus number of cycle of Zn(cur)O used for perylene degradation.

The half-life of ZnO and Zn(cur)O having different curcumin concentrations are given in Fig. 9c. It was found that when 0.5 mg of curcumin was used during synthesis of Zn(cur)O, the half-life reduced about 24% than that of bare ZnO. There was a slight increase in half-life when 1.0 and 1.5 mg of curcumin was incorporated. It is clear that the extent of curcumin conjugation during preparation of Zn(cur)O affects the removal capacity of Zn(cur)O in some way. The recyclability of Zn(cur)O was tested where the removal of perylene was studied in three consecutive cycles by reusing the same compound over and over again. As shown in Fig. 10, Zn(cur)O continued to remove PAHs in the three different cycles as studied. However, the half-life of perylene removal increased in each cycle as depicted in Fig. 10d indicating that the removal capacity of Zn(cur)O gets reduced hugely.

**Figure 11:**
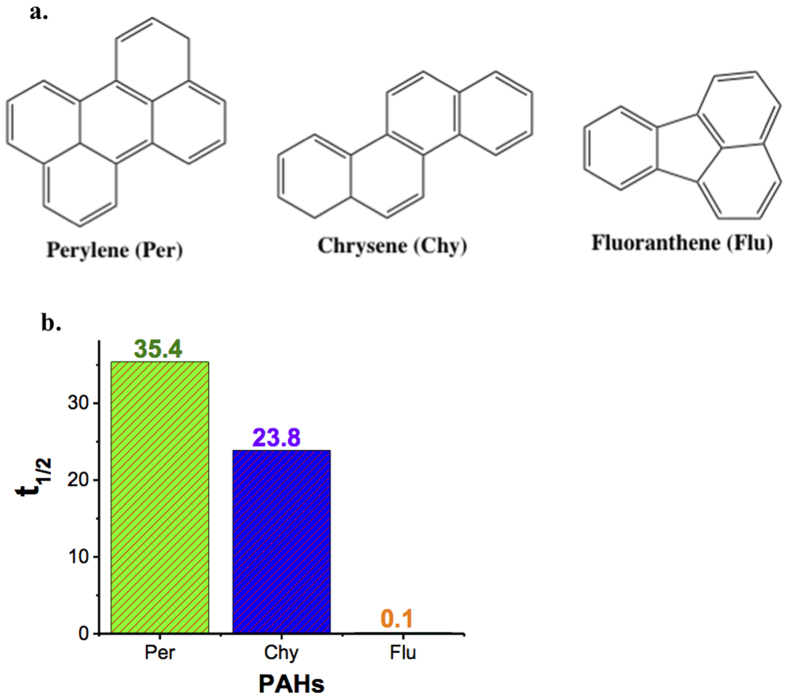
(**a**) Structure of perylene, chrysene and fluoranthene. (**b**) Comparison of half-life value during PAHs degradation by Zn(cur)O.

The removal of other PAHs like chrysene and fluoranthene (see Fig. 11a) was also tested by the suggested Zn(cur)O. In comparison to perylene, it was found that the half-life reduced appreciably by 33% for chrysene and remarkably by 99% for fluoranthene (see Fig. 11b). A possible explanation for this increase in the rate of removal when going from perylene to chrysene then to fluoranthene is the decreased number of aromatic rings or in other words the loss of conjugation that confers stability and resistance to the molecule. In fact, fluoranthene is a non-alternate PAH (presence of a 5 membered ring) whereas both perylene and chrysene are alternate PAHs, with perylene having more cyclic rings than chrysene. Thus, perylene and chrysene- being highly aromatic and more stable than fluoranthene- are expected to be less easily degraded compared to fluoranthene as confirmed by the results. Since ZnO is known to photo-catalytically degrade PAHs^16^,^42^, the discrepancy in removal of perylene, chrysene and fluoranthene suggest that the removal is due to photo-catalytic degradation. This is further evident from the earlier observation that curcumin conjugation increases the removal of perylene by ZnO, which could be due to the fact that curcumin allows exploiting the sun’s visible light energy and thus a faster degradation is expected.

It’s worth noting, that the relatively low Zn(cur)O loading of 167 mg/L showed good degradation ratios of 88% for relatively high concentrations of 12.6 mg/L perylene after 3.4 h, 93% for 11.4 mg/L fluoranthene in 10 minutes and 11.1 mg/L chrysene in 2.2 h. The light utilized is that of natural sunlight (i.e., both UV and visible light) as pointed out earlier. Photolysis is one of the major transformation processes affecting the fate of PAHs in the aquatic environment. Sunlight photo-alteration processes are well known to play an important role in the degradation of PAHs and other contaminants in water by generation of highly reactive intermediates, mainly hydroxyl radical (^*^OH), a powerful non-specific oxidant (Eº = 2.8 V)^14^. PAHs’ degradation could be due to direct photolysis since those compounds absorb light in the 200–400 nm range, which overlaps the emission spectrum of sunlight. However, under sunlight and with no photo-catalyst, the PAHs are found to degrade slowly compared with the reaction rates in the presence of photo-catalysts, especially ZnO. Vela *et al.*^14^ suggests 12% of 2–5 μg/L of fluoranthene remained after only 10 minutes with ZnO loading of 150 mg/L in comparison to 40% remaining after 50 minutes due to photolysis. Dass *et al.*^43^ tested the degradation of acenaphthene, anthracene, fluorene and naphthalene in aqueous suspension of TiO_2_ under UV and natural light irradiation, which came out to be highly effective due to the formation of hydroxyl and superoxide radicals. In another study, Ireland *et al.*^44^ obtained half-lives of 2.7 h in the case of anthracene and 9.2 h for fluoranthene using TiO_2_. Wen *et al.*^45^ found that phenanthrene (PAH of low solubility) in aqueous TiO_2_ suspensions under UV light irradiation was completely degraded after 40 minutes without significant effect of pH or amount of photocatalyst on degradation. The result of the current work is a 93% removal (7% remaining) of 11.4 mg/L fluoranthene with 167 mg/L Zn(cur)O loading after 10 minutes, which is similar to the result obtained when the photo-catalyst ZnO was present. This attributes the removal to the act of the photo-catalyst used, i.e., to photo-catalysis. The obtained results are quite promising if we take into consideration the moderate set of working conditions. For instance, the O_2_ requirement was provided by simply letting air go through the erlenmeyer’s opening, unlike other experiments where oxygen is purged continuously into the solution. Another important parameter, which has a significant effect on the rate, is the light intensity, which is considered weak in comparison to higher intensities used in studies. ZnO photocatalytic mechanism^46^ has been proven to be similar to that of TiO_2_^43^. We suggest the following working mechanism^47^:


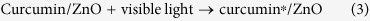



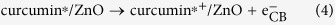



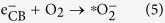



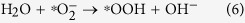



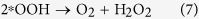



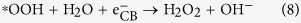



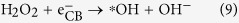



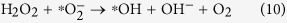



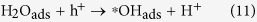



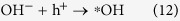



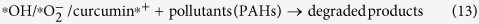


**Figure 12:**
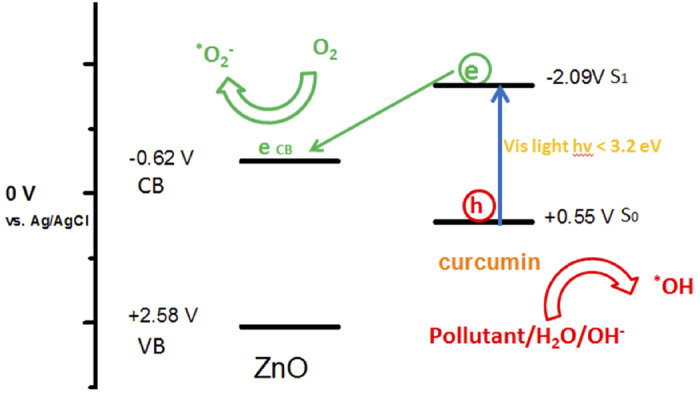
Schematic representation of the electron and hole transfer processes in the ZnO/curcumin systems.

For dye sensitization to be possible, the conduction band edge of the ZnO must be lower (more positive) than the LUMO (Lowest Unoccupied Molecular Orbital) level of the dye molecule, so that the excited electron of the dye can be injected into the semiconductor’s conduction band (CB). The reduction potential of the organic contaminant must be higher (more negative) than the HOMO (Highest Occupied Molecular Orbital) of the dye molecule. This condition is satisfied for the ZnO/curcumin system. Therefore, upon the photo-excitation of Zn(cur)O by a light with λ ≥ 445 nm and the consequent generation of electrons (e) and holes (h) in the conduction band (CB) and valence band (VB) respectively of curcumin, the excited electron is injected into the conduction band of ZnO (Eq. (4)). Then, the electron in the conduction band (*e*^−^_CB_) is transferred to molecular oxygen, leading to the formation of a series of radicals, which are active oxidizers capable of degrading organic pollutants in the system (PAHs) (Eqs (5, 6, 7, 8, 9, 10)). Subsequently, the pollutants can be degraded through a variety of paths (Eq. 13). Positive holes are thought to be trapped at the semiconductor’s surface and that they would readily react with surface absorbed water molecules or hydroxyl groups forming hydroxyl radicals (Eqs 11 and 12)^48^,^49^,^50^,^51^. It is also proposed that holes can directly oxidize adsorbed organic molecules resulting in the formation of organic radical cations^52^, which will subsequently undergo further oxidation and thus degradation. Figure 12 summarizes the mechanism.

## Conclusion

Curcumin conjugated ZnO nanostructures assembling into sub-micro grain-like structures were successfully synthesized, which are completely different from curcumin anchored on ZnO surfaces by physical adsorption process. The Zn(cur)O nanostructures materials were found to have wurtzite hexagonal crystal structure of ZnO and showed very good crystalline quality. The diffuse reflectance measurement suggested more impurities atom in Zn(cur)O compared to ZnO@cur. Chemically Zn(cur)O was more stable than ZnO@cur. Solid-state photoluminescence study confirmed increase in curcumin concentrations in Zn(cur)O suppressed visible emission of ZnO. Zn(cur)O could remove to a far extent high concentrations of perylene, fluoranthene, and chrysene marginally faster than ZnO depending on the extent of curcumin conjugation in Zn(cur)O. The removal was found to be faster for chrysene than perylene and extraordinary for flouranthene. The high rate of removal was related to photo-degradation and a mechanism has been proposed. The development of Zn(cur)O and the knowledge of the specific working parameters may open the door to a large-scale utilization of heterogeneous cost-effective photo catalysis via visible light to address water contamination and environmental pollution.

## Additional Information

**How to cite this article**: Moussawi, R. N. and Patra, D. Nanoparticle Self-Assembled Grain Like Curcumin Conjugated ZnO: Curcumin Conjugation Enhances Removal of Perylene, Fluoranthene, and Chrysene by ZnO. *Sci. Rep.* **6**, 24565; doi: 10.1038/srep24565 (2016).

## References

[b1] Lam S.-M., Sin J.-C., Abdullah A. Z. & Mohamed A. R. Degradation of Wastewaters Containing Organic Dyes Photocatalysed by Zinc Oxide: A Review. Desal. Water Treatm. 41, 131–169 (2012).

[b2] Rengarajan T. *et al.* Exposure to Polycyclic Aromatic Hydrocarbons with Special Focus on Cancer. Asian Pacific J. Trop. Biomed. 5, 182–189 (2015).

[b3] Baek S., Goldstone M., Kirk P., Lester J. & Perry R. Phase Distribution and Particle Size Dependency of Polycyclic Aromatic Hydrocarbons in the Urban Atmosphere. Chemosph. 22, 503–520 (1991).

[b4] Grice K. *et al.* New Insights into the Origin of Perylene in Geological Samples. Geochim. et Cosmochim. Acta 73, 6531–6543 (2009).

[b5] Brun G. L., Vaidya O. C. & Léger M. G. Atmospheric Deposition of Polycyclic Aromatic Hydrocarbons to Atlantic Canada: Geographic and Temporal Distributions and Trends 1980-2001. Environ. Sci. Technol. 38, 1941–1948 (2004). 15112792 10.1021/es034645l

[b6] Clemons J. *et al.* Evidence of Estrogen-and TCDD-like Activities in Crude and Fractionated Extracts of PM10 Air Particulate Material Using *in vitro* Gene Expression Assays. Environ. Sci. Technol. 32, 1853–1860 (1998).

[b7] Liu X. *et al.* Compositional Fractionation of Polycyclic Aromatic Hydrocarbons (PAHs) in Mosses from the Northern Slope of Nanling Mountains, South China. Atmosph. Environ. 39, 5490–5499 (2005).

[b8] Freeman D. J. & Cattell F. C. Woodburning as a Source of Atmospheric Polycyclic Aromatic Hydrocarbons. Environ. Sci. Technol. 24, 1581–1585 (1990).

[b9] Shi Z. *et al.* Contamination of Rivers in Tianjin, China by Polycyclic Aromatic Hydrocarbons. Environ. Pollut. 134, 97–111 (2005). 15572228 10.1016/j.envpol.2004.07.014

[b10] Tan Y. L., Quanci J. F., Borys R. D. & Quanci M. J. Polycyclic Aromatic Hydrocarbons in Smoke Particles from Wood and Duff Burning. Atmosph. Environ. A: General Topics 26, 1177–1181 (1992).

[b11] Nielsen T., Seitz B. & Ramdahl T. Occurrence of Nitro-PAH in the Atmosphere in a Rural Area. Atmosph. Environ. 18, 2159–2165 (1984).

[b12] Mumford J. L. *et al.* Indoor Air Pollutants from Unvented Kerosene Heater Emissions in Mobile Homes: Studies on Particles, Semivolatile Organics, Carbon Monoxide, and Mutagenicity. Environ. Sci. Techno. 25, 1732–1738 (1991).

[b13] Traynor G. W. *et al.* Selected Organic Pollutant Emissions from Unvented Kerosene Space Heaters. Environ. Sci. Techno. 24, 1265–1270 (1990).

[b14] Vela N., Martínez-Menchón M., Navarro G., Pérez-Lucas G. & Navarro S. Removal of Polycyclic Aromatic Hydrocarbons (PAHs) from Groundwater by Heterogeneous Photocatalysis under Natural Sunlight. J. Photochem. Photobiol. A: Chemistry 232, 32–40 (2012).

[b15] Sakthivel S. *et al.* Solar Photocatalytic Degradation of Azo Dye: Comparison of Photocatalytic Efficiency of ZnO and TiO_2_. *Solar Ener*. Mater. Solar Cells 77, 65–82 (2003).

[b16] Kou J. *et al.* Photocatalytic Degradation of Polycyclic Aromatic Hydrocarbons in GaN: ZnO Solid Solution-Assisted Process: Direct Hole Oxidation Mechanism. J. Molecul. Catal. A: Chemical 325, 48–54 (2010).

[b17] Buddee S., Wongnawa S., Sriprang P. & Sriwong C. Curcumin-Sensitized TiO_2_ for Enhanced Photodegradation of Dyes under Visible Light. J. Nanopart. Res. 16, 1–21 (2014).

[b18] Goel A., Kunnumakkara A. B. & Aggarwal B. B. Curcumin as “Curecumin”: From Kitchen to Clinic. Biochem. Pharmacol. 75, 787–809 (2008). 17900536 10.1016/j.bcp.2007.08.016

[b19] Aggarwal B. B. & Harikumar K. B. Potential Therapeutic Effects of Curcumin, the Anti-inflammatory Agent, Against Neurodegenerative, Cardiovascular, Pulmonary, Metabolic, Autoimmune and Neoplastic Diseases. Internat. J. Biochem. Cell Biol. 41, 40–59 (2009). 10.1016/j.biocel.2008.06.010 PMC2637808 18662800

[b20] Srivastava R. M., Singh S., Dubey S. K., Misra K. & Khar A. Immunomodulatory and Therapeutic Activity of Curcumin. Intern. Immunopharmacol. 11, 331–341 (2011). 10.1016/j.intimp.2010.08.014 20828642

[b21] Ganesh T. *et al.* Photoactive Curcumin-Derived Dyes with Surface Anchoring Moieties Used in ZnO Nanoparticle-based Dye-Sensitized Solar Cells. Mater. Chem. Phys. 123, 62–66 (2010).

[b22] Patra D. & Barakat C. Unique Role of Ionic Liquid [bmin][BF4] During Curcumin–Surfactant Association and Micellization of Cationic, Anionic and Non-ionic Surfactant Solutions. Spectrochim. Acta A 79, 1823–1828 (2011). 10.1016/j.saa.2011.05.064 21684197

[b23] Patra D., El Khoury E., Ahmadieh D., Darwish S. & Tafech R. M. Effect of Curcumin on Liposome: Curcumin as a Molecular Probe for Monitoring Interaction of Ionic Liquids with 1,2‐Dipalmitoyl‐sn‐Glycero‐3‐Phosphocholine Liposome. Photochem. Photobiol. 88, 317–327 (2012). 22191485 10.1111/j.1751-1097.2011.01067.x

[b24] El Khoury E. & Patra D. Ionic Liquid Expedites Partition of Curcumin into Solid Gel but Discourages into Liquid Crystalline Phases of 1,2-Dimyristoyl-sn-Glycero-3-Phosphocholine Liposome. J. Phys. Chem. B 117, 9699–9708 (2013). 23895644 10.1021/jp4061413

[b25] Patra D., Aridi R. & Bouhadir K. Fluorometric Sensing of DNA Using Curcumin Encapsulated Nanoparticles Assembled Microcapsules from Poly(Diallylammonium Chloride-co-Sulfur Dioxide). Microchim. Acta 180, 59–64 (2013).

[b26] Mouslmani M., Bouhadir K. H. & Patra D. Poly (9-(2-Diallylaminoethyl)Adenine HCl-co-Sulfur Dioxide) Deposited on Silica Nanoparticles Constructs Hierarchically Ordered Nanocapsules: Curcumin Conjugated Nanocapsules as a Novel Strategy to Amplify Guanine Selectivity Among Nucleobases. Biosens. Bioelectron. 68, 181–188 (2015). 25569875 10.1016/j.bios.2014.12.036

[b27] Zhou S.-S. *et al.* Synthesis, Optical Properties and Biological Imaging of the Rare Earth Complexes with Curcumin and Pyridine. J. Mater. Chem. 22, 22774–22780 (2012).

[b28] Ciszewski A., Milczarek G., Lewandowska B. & Krutowski K. Electrocatalytic Properties of Electropolymerized Ni (II) Curcumin Complex. Electroanaly. 15, 518–523 (2003).

[b29] Renfrew A. K., Bryce N. S. & Hambley T. W. Delivery and Release of Curcumin by a Hypoxia-Activated Cobalt Chaperone: A XANES and FLIM Study. Chem. Sci. 4, 3731–3739 (2013).

[b30] El Khoury E., Abiad M., Kassaify Z. G. & Patra D. Green Synthesis of Curucmin Conjugated Nanosilver for the Applications in Nucleic Acid Sensing and Anti-bacterial Activity. Colloids Surf. B: Biointerf. 117, 274–280 (2015). 10.1016/j.colsurfb.2015.01.050 25687098

[b31] Moussawi R. N. & Patra D. Synthesis of Au Nanorods through Pre-Reduction with Curcumin: Preferential Enhancement of Au Nanorods Formation Prepared from CTAB Capped over Citrate Capped Au Seeds. J. Phys. Chem. C 119, 19458–19468 (2015).

[b32] Caruso F. *et al.* Ruthenium–Arene Complexes of Curcumin: X-ray and Density Functional Theory Structure, Synthesis, and Spectroscopic Characterization, *in vitro* Antitumor Activity, and DNA Docking Studies of (p-Cymene) Ru (Curcuminato) Chloro. J. Medic. Chem. 55, 1072–1081 (2012). 10.1021/jm200912j 22204522

[b33] Khalil M. I., Al-Qunaibit M. M., Al-zahem A. M. & Labis J. P. Synthesis and Characterization of ZnO Nanoparticles by Thermal Decomposition of a Curcumin Zinc Complex. Arab. J. Chem. 7, 1178–1184 (2014).

[b34] Begum G., Manorama S. V., Singh S. & Rana R. K. Morphology-Controlled Assembly of ZnO Nanostructures: A Bioinspired Method and Visible Luminescence. Chem. Eur. J. 14, 6421–6427 (2008). 18528921 10.1002/chem.200800129

[b35] Mishra K., Srivastava R. K. & Prakash S. G. Photoluminescence and Photoconductivity Studies of ZnO Nanoparticles Prepared by Solid State Reaction Method. J. Mater. Sci.: Mater. Electron. 24, 125–134 (2013).

[b36] El-Rahman S. N. A. & Al-Jameel S. S. Protection of Curcumin and Curcumin Nanoparticles Against Cisplatin Induced Nephrotoxicity in Male Rats. Sch. Acad. J. Biosci. 2, 214–223 (2014).

[b37] Lu Z., Zhou J., Wang A., Wang N. & Yang X. Synthesis of Aluminium-Doped ZnO Nanocrystals with Controllable Morphology and Enhanced Electrical Conductivity. J. Mater. Chem. 21, 4161–4167 (2011).

[b38] Kolev T. M., Velcheva E. A., Stamboliyska B. A. & Spiteller M. DFT and Experimental Studies of the Structure and Vibrational Spectra of Curcumin. Int. J. Quant. Chem. 102, 1069–1079 (2005).

[b39] Ngo-Duc T., Singh K., Meyyappan M. & Oye M. M. Vertical ZnO Nanowire Growth on Metal Substrates. Nanotechnol. 23, 194015 (2012). 10.1088/0957-4484/23/19/194015 22538243

[b40] Berger L. I. Semiconductor Materials, FL, USA (CRC Press, Boca Raton, 1997).

[b41] Benramache S., Benhaoua B. & Bentrah H. Preparation of Transparent, Conductive ZnO: Co and ZnO: In Thin Films by Ultrasonic Spray Method. J. Nanostruct. Chem. 3, 54 (2013).

[b42] Martinez-Menchon M., Navarro G., Perez-Lucas G., Navarro S. & Vela N. Removal of Polycyclic Aromatic Hydrocarbons (PAHs) from Ground Water by Heterogeneous Photocatalyst under Natural Light. J. Photochem. Photobiol. A: Chem. 232, 32–40 (2012).

[b43] Dass S., Muneer M. & Gopidas K. Photocatalytic Degradation of Wastewater Pollutants. Titanium-Dioxide-Mediated Oxidation of Polynuclear Aromatic Hydrocarbons. J. Photochem. Photobiol. A: Chem. 77, 83–88 (1994).

[b44] Ireland J. C., Dávila B., Moreno H., Fink S. K. & Tassos S. Heterogeneous Photocatalytic Decomposition of Polyaromatic Hydrocarbons Over Titanium Dioxide. Chemosph. 30, 965–984 (1995).

[b45] Wen S., Zhao J., Sheng G., Fu J. & Peng P. A. Photocatalytic Reactions of Pyrene at TiO_2_/Water Interfaces. Chemosph. 50, 111–119 (2003). 10.1016/s0045-6535(02)00420-4 12656236

[b46] Dindar B. & Içli S. Unusual Photoreactivity of Zinc Oxide Irradiated by Concentrated Sunlight. J. Photochem. Photobiol. A: Chem. 140, 263–268 (2001).

[b47] Theurich J. *et al.* Photocatalytic Degradation of Naphthalene and Anthracene: GC-MS Analysis of the Degradation Pathway. Res. Chem. Intermed. 23, 247–274 (1997).

[b48] Bard A. J., Parsons B. & Jordon J. (eds.) Standard Potentials in Aqueous Solution. New York (Dekker, 1985).

[b49] Anpo M., Shima T. & Kubokawa Y. ESR and Photoluminescence Evidence for the Photocatalytic Fromation of Hydroxyl Radicals on Small TiO_2_ Particles. Chem. Lett. 14, 1799–1802 (1985).

[b50] Lepore G. P., Langford C. H., Vichova J. & Vlček A. Jr Photochemistry and Picosecond Absorption Spectra of Aqueous Suspensions of a Polycrystalline Titanium Dioxide Optically Transparent in the Visible Spectrum. J. Photochem. Photobiol. A: Chem. 75, 67–75 (1993).

[b51] Jaeger C. D. & Bard A. J. Spin Trapping and Electron Spin Resonance Detection of Radical Intermediates in the Photodecomposition of Water at Titanium Dioxide Particulate Systems. J. Phys. Chem. 83, 3146–3152 (1979).

[b52] Fox M. A., Chen C. C. & Younathan J. N. N. Oxidative Cleavage of Substituted Naphthalenes Induced by Irradiated Semiconductor Powders. J. Org. Chem. 49, 1969–1974 (1984).

